# Tracheostomy is associated with decreased hospital mortality after moderate or severe isolated traumatic brain injury

**DOI:** 10.1007/s00508-016-1004-y

**Published:** 2016-05-25

**Authors:** David Marek Baron, Helene Hochrieser, Philipp G. H. Metnitz, Walter Mauritz

**Affiliations:** Department of Anesthesiology, General Intensive Care and Pain Management, Medical University of Vienna, Vienna, Austria; Department of Medical Statistics, Medical University of Vienna, Vienna, Austria; Department of Anesthesiology and Intensive Care Medicine, Medical University of Graz, Graz, Austria; Department of Anesthesia and Critical Care Medicine, Trauma Hospital “Lorenz Boehler”, Vienna, Austria

**Keywords:** Isolated traumatic brain injury, Tracheostomy, Hospital mortality, Outcome

## Abstract

**Background:**

Data regarding the impact and timing of tracheostomy in patients with isolated traumatic brain injury (TBI) are ambiguous. Our goal was to evaluate the impact of tracheostomy on hospital mortality in patients with moderate or severe isolated TBI.

**Materials and Methods:**

We performed a retrospective cohort analysis of data prospectively collected at 87 Austrian intensive care units (ICUs). All patients continuously admitted between 1998 and 2010 were evaluated for the study. In total, 4,735 patients were admitted to ICUs with isolated TBI. Of these patients, 2,156 had a moderate or severe TBI (1,603 patients were endotracheally intubated only, 553 patients underwent tracheostomy). Epidemiological data (trauma severity, treatment, and outcome) of the two groups were compared.

**Results:**

Patients with moderate or severe isolated TBI undergoing tracheostomy had a similar Glasgow Coma Scale score, median (interquartile range): 6 (3–8) vs 6 (3–8); *p* = 0.90, and Simplified Acute Physiology Score II, 45 (37–54) vs 45 (35–56); *p* = 0.86, compared with intubated patients not undergoing tracheostomy. Furthermore, patients undergoing tracheostomy exhibited higher Abbreviated Injury Scale Head scores and had a longer ICU stay for survivors, 30 (22–42) vs 9 (3–17) days; *p* < 0.0001). In contrast, risk-adjusted mortality was lower in patients undergoing tracheostomy compared with patients who remained intubated, observed-to-expected mortality ratio (95 % confidence interval): 0.62 (0.53–0.72) vs 1.00 (0.95–1.05) respectively.

**Conclusions:**

Despite the greater severity of head injury, patients with isolated TBI who underwent tracheostomy had a lower risk-adjusted mortality than patients who remained intubated. Reasons for this difference in outcome may be multifactorial and require further investigation.

## Introduction

Following admission to the intensive care unit (ICU) patients with moderate or severe traumatic brain injury (TBI; defined as a Glasgow Coma Scale [GCS] score ≤12) usually require prolonged analgesia, sedation, and ventilation. Many of these patients arrive at the ICU with an endotracheal tube in place. During their ICU stay, some of these patients require a tracheostomy. At present, data are ambiguous as to whether or not tracheostomy has any impact on outcome in patients with moderate or severe TBI.

Almost 20 years ago Lesnik et al. [1] recommended early tracheostomy within 4 days of the injury for patients with blunt multiple trauma. The authors found that late tracheostomy was associated with longer duration of ventilation and higher rates of pulmonary infections. This finding was supported by D’Amelio et al. [2], who studied 43 patients with severe TBI (defined as an Abbreviated Injury Scale [AIS] score for the head region >2). The authors reported a shorter duration of ventilation, and shorter ICU and hospital length of stay (LOS) in trauma patients undergoing tracheostomy. Comparable results have been published by Kluger et al. [3], Teoh et al. [4], and Ahmed et al. [5]. Other authors, however, found no significant benefits of early tracheostomy in patients with TBI [6]. Stocchetti et al. [7] were the first to point out that the procedure may increase intracranial pressure (ICP) in patients with brain damage. These results have been supported by further studies [8, 9]. Kocaeli et al. [9] studied the effects of tracheostomy upon ICP during early (within 7 days) or late (after 7 days) tracheostomy in patients with significant brain pathological conditions (GCS < 8). In patients with early tracheostomy, ICP nearly doubled during the procedure, whereas ICP increased only 30 % during late tracheostomy. Conversely, Milanchi et al. found no significant increases in ICP during tracheostomy in patients with TBI [10]. Thus, at present, it is unclear whether tracheostomy is beneficial for patients with TBI.

The goal of the current study was to investigate whether tracheostomy was beneficial in patients with isolated TBI. We analyzed data from a large cohort of patients collected at 87 Austrian ICUs. We report that patients with moderate or severe isolated TBI undergoing tracheostomy had a lower risk-adjusted mortality compared with patients who remained intubated.

## Materials and Methods

The study protocol was submitted to and approved by the institutional ethics committee. Since no interventions were performed and no individual data were analyzed, the need for informed consent was waived.

Data were collected by the Austrian Center for Documentation and Quality Assurance in Intensive Care Medicine (ASDI), a non-profit organization that has established an ICU database and benchmarking project in Austria. The prospectively collected data included socio-demographic parameters such as age, sex, and chronic conditions (comorbidities); the reason for ICU admission according to a list of medical and surgical diagnoses [11]; the severity of illness and of trauma according to the GCS score, Simplified Acute Physiology Score (SAPS) II, and Injury Severity Score (ISS) [12–14], all determined at admission. For patients sedated at admission, the GCS score obtained immediately before sedation was used. In addition, the level of care provided, as measured by the Therapeutic Intervention Scoring System (TISS-28) [15], was recorded daily. The length of ICU and hospital stays and the status at ICU and hospital discharge (survival/death) were recorded.

To assess the reliability of data collection, we sent an independent observer to each unit to obtain SAPS II data from the clinical charts of a random sample of patients. Variance-component analyses with the random factors “units,” “patients within units,” and “observers within units” were performed. As described previously, the main source of variation for most of the variables was the variability between “patients within units,” whereas “units” did not substantially contribute as an additional source of variation. Overall, the results indicated an excellent grade of agreement [16]. To assess the completeness of the documentation, we calculated the number of missing parameters for the SAPS II and achieved satisfactory results. More details have been reported elsewhere [16].

**Fig. 1:**
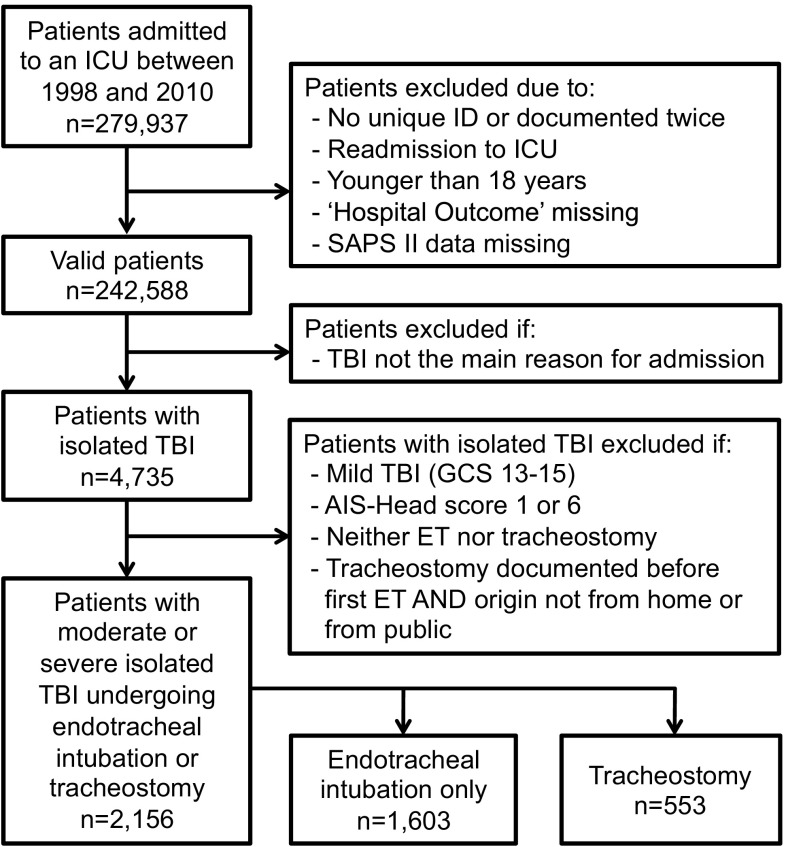
Flow diagram of the study. *AIS* Abbreviated Injury Scale, *ET* endotracheal tube, *GCS* Glasgow Coma Scale, *ICU* intensive care unit, *SAPS* Simplified Acute Physiology Score, *TBI* traumatic brain injury

Between 1998 and 2010, a total of 279,937 patients were admitted to 87 Austrian ICUs (Fig. 1). Patients without a unique ID and those documented twice were excluded from analysis (*n* = 366). For patients who were admitted more than once (*n* = 19,426), only the first admission was included. Patients who were younger than 18 years (*n* = 5,386), those with records that lacked an entry in the field “hospital outcome” (*n* = 2,108) and those without a valid SAPS II (*n* = 10,063) were also excluded, leaving a cohort of 242,588 patients. From this cohort, in TBI was the main reason for admission in 6,871 patients. Of these, 2,136 patients were excluded because regions other than the head were affected by trauma (e. g., abdomen, thorax, or extremities), leaving 4,735 patients with isolated TBI. Finally, patients without endotracheal intubation or tracheostomy during their ICU stay (*n* = 1,957), those with mild TBI, defined by a GCS between 13 and 15 (*n* = 572), and patients with an AIS head score of 1 or 6 (*n* = 50) were excluded. Thus, the final study cohort included a total of 2,156 patients with moderate or severe isolated TBI; 1,603 patients were only endotracheally intubated, whereas 553 patients had additionally undergone tracheostomy. Because of the design of the database, a breakdown into surgical and percutaneous dilatational tracheostomies was not possible.

### Statistical analysis

Statistical analysis was performed using SAS software version 9.2 (SAS Institute, Cary, NC, USA) and R 2.14.1. For tests of statistical significance, ANOVA was performed for normally distributed data. The Kruskal–Wallis test was used if data were not normally distributed. Furthermore, the Chi-squared test was used when appropriate. A *p* value of <0.05 was considered significant. Unless otherwise specified, descriptive results are expressed as median and first and third quartiles respectively. Observed-to-expected mortality ratios as surrogates for risk-adjusted mortality were calculated by dividing the number of observed deaths per group by the number of SAPS II-predicted deaths per group, and are indicated with their corresponding 95 % confidence intervals (CIs).

To evaluate factors associated with a tracheostomy after admission to the ICU, we applied methods for cumulative incidences (R-functions cuminc – package cmprsk). A competing risk regression model to investigate the impact of different variables on receiving a tracheostomy after ICU admission was calculated (R-functions crr – package cmprsk) [17]. The co-variables used were the SAPS II, the AIS head score, and the number of TBI cases per year as a unit-specific influence variable. Results are depicted as cumulative incidences with 95 % CIs.

## Results

### Demographic data and trauma severity of the study population

Data on demographics and on trauma severity are reported in Table 1. Of the 2,156 patients with moderate or severe isolated TBI, 553 patients (26 %) received a tracheostomy, whereas 1,603 patients (74 %) remained endotracheally intubated. Patients undergoing tracheostomy were older and displayed an AIS head score of 4 more often than intubated patients. In contrast, an AIS head score of 2 was more frequent in intubated patients who did not undergo tracheostomy. The incidence of patients with an AIS head score of 3 and 5 and the incidence of comorbidities, GCS scores, and SAPS II in the two groups were not different.

**Table 1:** Demographic data and trauma severity of the study population

	Cohort			*p* value (ET vs TR)	
	ET	ET, then TR	Total	ANOVA	Kruskal–Wallis
Patients, n (%)	1,603 (74)	553 (26)	2,156 (100)		
Age (years; median IQR)	58 (40–74)	62 (47–75)	59 (42–74)		0.004
Sex					
Male, *n* (%)	1,167 (73)	394 (71)	1,561 (72)	0.49	
Female, *n* (%)	434 (27)	158 (29)	592 (28)	0.49	
AIS head score, *n* (%)					
2	85 (5.3)	8 (1.5)	93 (4.3)	< 0.001	
3	300 (18.7)	88 (15.9)	388 (18.0)	0.14	
4	636 (39.7)	257 (46.5)	893 (41.4)	0.005	
5	582 (36.3)	200 (36.1)	782 (36.3)	0.95	
GCS score, median (IQR)	6 (3–8)	6 (3–8)	6 (3–8)		0.90
SAPS II, median (IQR)	45 (36–56)	45 (35–56)	45 (37–54)		0.86
No comorbidity, *n* (%)	1,146 (72)	378 (68)	1,524 (71)	0.16	
Mortality predicted by SAPS II, %	40	38	39		0.86

*AIS*  Abbreviated Injury Scale, *ANOVA* analysis of variance, *CI* confidence interval, *ET* endotracheal tube, *GCS* Glasgow Coma Scale score, *IQR* interquartile range, *SAPS* Simplified Acute Physiology Score, *TISS* Therapeutic Intervention Scoring System, *TR* tracheostomy

### Treatment and outcome in patients with moderate or severe isolated TBI

Data regarding treatment and outcome are depicted in Table 2. Tracheostomy was usually performed in the second or third week after sustaining the injury. Patients receiving a tracheostomy underwent neurosurgery more frequently, exhibited a higher level of treatment (as indicated by more TISS-28 points per treatment day), and had a longer ICU stay than intubated patients. The ICU and overall hospital mortality were higher in patients who did not receive a tracheostomy, whereas post-ICU mortality was higher in patients undergoing tracheostomy. Risk-adjusted mortality (observed-to-expected mortality ratio) was significantly lower in patients undergoing tracheostomy (0.62 [0.53–0.72]) compared with patients who remained intubated (1.00 [0.95–1.05]).

**Table 2:** Treatment and outcome

	Cohort			*p* value (ET vs TR)	
	ET	ET, then TR	Total	ANOVA	Kruskal–Wallis
Patients, *n* (%)	1,603 (74)	553 (26)	2,156 (100)		
Day of tracheostomy, median (IQR)		12 (7–19)			
Surgical treatment of TBI, *n* (%)	962 (60)	402 (73)	1,364 (63)		< 0.0001
TISS-28 points per patient, median (IQR)	237 (96–508)	1,062 (714–1,456)	376 (130–834)		< 0.0001
TISS-28 points per patient per day, median (IQR)	36 (31–41)	37 (34–40)	36 (32–41)		< 0.0001
Length of ICU stay, median (IQR)	7 (3–14)	29 (19–41)	11 (4–23)		< 0.0001
Length of ICU stay survivors, median, (IQR)	9 (3–17)	30 (22–42)	15 (5–28)		< 0.0001
Outcome, *n* (%)					
ICU deaths	578 (36)	72 (13)	650 (30)	< 0.0001	
Post-ICU deaths	58 (4)	60 (11)	118 (6)	< 0.0001	
Total hospital deaths	636 (40)	132 (24)	768 (36)	< 0.0001	
Observed vs expected deaths ratio (CI)	1.00 (0.95–1.05)	0.62 (0.53–0.72)	0.90 (0.86–0.95)		

*ICU* intensive care unit, *TBI* traumatic brain injury

### Incidence of discharge, death, or tracheostomy in patients with isolated TBI

**Fig. 2:**
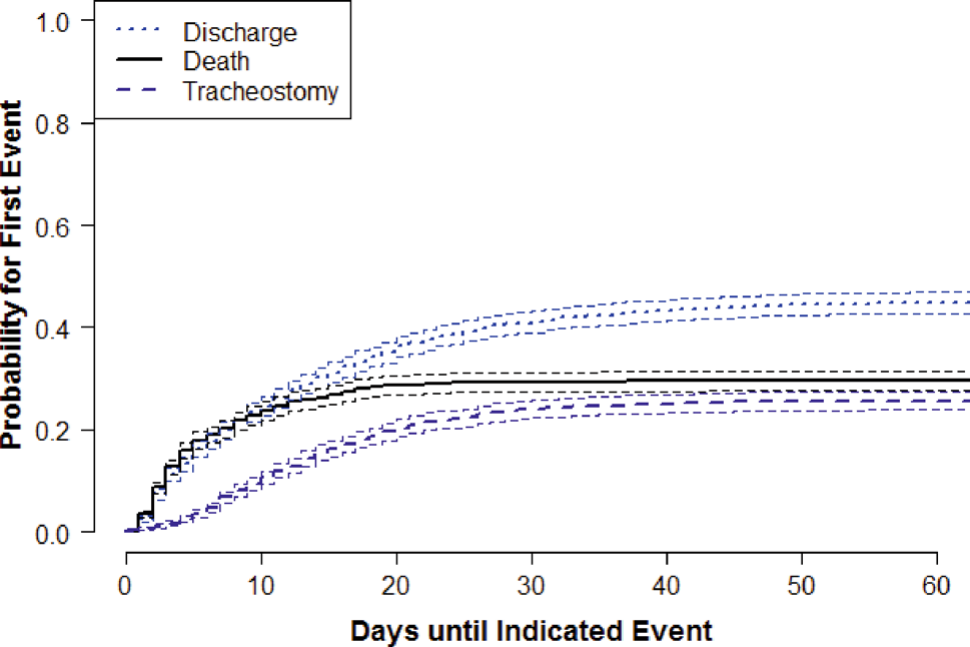
Cumulative incidences and confidence intervals for three possible disjoint events (discharge, death, tracheostomy) in patients with moderate or severe isolated TBI (*n* = 2,156). For each time point the proportion of patients who experienced one of the events (as the first event) is shown. *Dotted line*: patients discharged first (without undergoing tracheostomy); *dashed line*: patients undergoing tracheostomy; *solid line*: patients who died before receiving a tracheostomy

Cumulative incidences and confidence intervals for three possible distinct events (discharge, death, and tracheostomy) in patients with moderate or severe isolated TBI (*n* = 2,156) are illustrated in Fig. 2. For each time point, the proportion of patients who experienced at least one of these events is shown. After approximately 30 days the curves reach a plateau, because any one of the three events has taken place in every patient. The diagram illustrates that many patients who did not undergo tracheostomy died or were discharged within the first week of admission, and that most patients received the tracheostomy within the second or third week of admission to the ICU.

Multivariate analysis for the cumulative incidence of tracheostomy revealed a marked impact of the AIS head score (1.29 [1.17–1–42], *p* < 0.001). Thus, patients with a higher AIS head score had a greater probability of undergoing tracheostomy. The number of head-trauma cases per ICU per year (0.99 [0.98–1.00], *p* = 0.12) and the SAPS II (1.00 [0.99–1.01], = 0.52) did not show a noticeable impact on the probability of undergoing a tracheostomy. However, we observed a correlation between SAPS II and the AIS head score (R = 0.52, *p* < 0.001), indicating that the AIS head score covers the influence of the SAPS II.

### Incidence of discharge or death in patients with isolated TBI undergoing tracheostomy

**Fig. 3:**
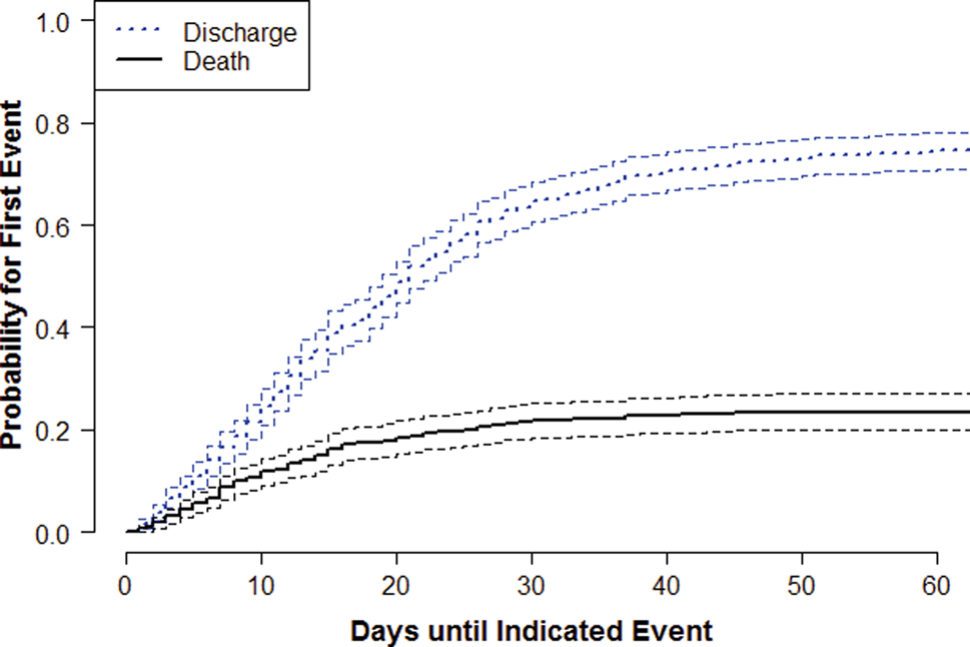
Cumulative incidence and confidence intervals for two possible disjoint events (discharge, death) in patients with moderate or severe isolated TBI undergoing tracheostomy (*n* = 553). *Dotted line*: patients discharged alive; *solid line*: patients who died during the hospital stay

The cumulative incidence and confidence intervals of in-hospital death or discharge from the ICU in the subset of patients undergoing tracheostomy (*n* = 553) are depicted in Fig. 3. Multivariate analysis revealed a marked influence of SAPS II (HR 1.03 [95 % CI 1.02–1.05], *p* < 0.001) on the cumulative incidence of in-hospital mortality. The results also show a negative association between time (admission to the ICU until tracheostomy) and hospital mortality (HR 0.97 [95 % CI 0.95–0.99], *p* = 0.003), indicating that late tracheostomy might be associated with improved outcome. The AIS head score, the number of head-trauma cases per ICU per year, and TISS-28 points per patient per day from admission until tracheostomy did not have an impact on mortality in patients undergoing tracheostomy (data not shown).

## Discussion

In the current study, we report that tracheostomy was associated with decreased ICU and hospital mortality in patients with moderate or severe isolated TBI compared with patients who remained intubated. Our results suggest that tracheostomy may improve outcome in patients with isolated TBI, as indicated by the lower risk-adjusted mortality rates for these patients. To the best of our knowledge this is the largest study comparing the effects of endotracheal intubation and tracheostomy on outcome in patients with isolated TBI.

Hukkelhoven et al. reported that the odds of a poor outcome increased by 40 to 50 % per 10 years of life in patients with closed TBI [18]. In our study, patients undergoing tracheostomy were 10 years older than those who remained endotracheally intubated. Thus, these older patients undergoing tracheostomy were supposed to have a worse outcome. However, tracheostomy was associated with improved outcome in patients with moderate and severe TBI in our analysis. In addition, neurosurgery (i. e., craniotomy or ICP monitoring) was performed more frequently in patients undergoing tracheostomy. This finding may indicate more severe TBI, or simply that neurosurgery was expected to be beneficial in these patients.

Optimal management of the airway is crucial for outcome in patients with TBI. Potential benefits of a tracheostomy include improved patient comfort, better patient mobility, and improved weaning by the reduction of respiratory resistance, the facilitation of pulmonary toilet, and decreased sedation requirements [19]. Furthermore, tracheostomy is associated with a reduced incidence of ventilator-associated pneumonia [20]. Previous studies reported that the sickest patients require longer ventilator times and are therefore more likely to undergo a tracheostomy [21]. Similar results were observed in our study. Patients undergoing tracheostomy had higher AIS head scores and had a longer ICU LOS than patients who remained intubated. As a consequence, weaning from mechanical ventilation may have been easier in intubated patients, leading to earlier discharge from ICU in these patients. In addition, as illustrated in Fig. 2, most deaths occurred within the first week of injury. Thus, the shorter ICU LOS in intubated patients can in part be explained by the fact that the sickest patients in our study probably died before they could receive a tracheostomy.

Several studies have attempted to define the ideal time to perform a tracheostomy. Some of these studies compared early tracheostomy with prolonged endotracheal intubation [22, 23], whereas others evaluated the effects of early versus late tracheostomy [2–5, 9, 24]. As early as 1992, Lesnik et al. reported that a tracheostomy performed within the first 4 days of injury facilitated weaning from the ventilator in patients who had sustained blunt, multiple organ trauma [1]. Two years later, D’Amelio et al. identified patients with TBI and multiple trauma to have a shorter duration of mechanical ventilation, as along with decreased ICU and hospital LOS if the tracheostomy was performed within 7 days of the injury [2]. Kluger et al. suggested reduced rates of septic complications when tracheostomy was performed within 3 days of the injury in trauma patients [3]. More recent studies examining similar patient populations reported a reduced LOS in patients undergoing early tracheostomy [4, 5]. In 2012, Wang et al. demonstrated that early tracheostomy performed by day 10 after severe head injury may contribute to a shorter duration of ICU LOS, a lower incidence of Gram-negative microorganism-related nosocomial pneumonia, and a shorter duration of antibiotic use [25]. Similarly, Rizk et al. reported that early tracheostomy resulted in a better overall clinical outcome in TBI patients with associated injuries in at least one other body region, especially when performed in those patients with a reasonable chance of survival [24]. Recently, Alali et al. reported that early tracheostomy within 8 days of injury was associated with a shorter duration of mechanical ventilation and reduced ICU and hospital LOS, but did not reduce hospital mortality [26].

In contrast, a meta-analysis published by Wang et al. failed to show the benefits of early tracheostomy in critically ill patients [27]. Early tracheostomy did not reduce the incidence of ventilator-associated pneumonia [28], the duration of mechanical ventilation or sedation, and was not associated with a shorter ICU or hospital LOS. The Cochrane Collaboration published a systematic review in 2012, which concluded that there was no specific information about any subgroup or individual characteristics potentially associated with better outcome with either early or late tracheostomy in critically ill patients [29]. Similarly, the “TracMan randomized trial” failed to show any benefit of early tracheostomy on clinical outcome [30]. In our study, tracheostomy was mostly performed in the second and third weeks after injury, meeting the definition of late tracheostomy in most studies.

Only part of the damage to the brain during TBI occurs at the moment of impact. Secondary insults can worsen the initial damage in subsequent days. Data published since the 1970s indicate that significant reductions in mortality and morbidity can be achieved in patients with severe TBI by using management protocols, which include monitoring of the ICP. In addition, the frequency of ICP monitoring in trauma centers has been reported to be associated with improved outcome [31–34]. Changes in ICP during tracheostomy and varying increases in ICP at different time points of the ICU stay have been reported. Kocaeli et al. [9] and Milanchi et al. [10] observed only insignificant rises in ICP during percutaneous tracheostomy in neurosurgical patients. Stocchetti et al., however, reported a significant increase in ICP while performing the tracheostomy [7]. Furthermore, Imperiale et al. reported a trend toward a transient ICP increase during percutaneous tracheostomy without a reduction in cerebral perfusion pressure [8]. Thus, performing a tracheostomy may cause intracranial hypertension, which may be more harmful during the first days after TBI. As most our patients received their tracheostomy rather late, negative effects of the procedure could perhaps have been mitigated.

Our study has several limitations. The database itself provided only limited information with regard to TBI. We have no information on the cause of TBI, on the different components of the GCS score, or on radiological findings, which would have been necessary to calculate the Marshall score. Furthermore, the database does not provide information on ICP or cerebral perfusion pressure, on the clinical management of increased ICP, or on detailed surgical interventions. As with most databases, the individual decision-finding of the treating physicians cannot be illustrated. However, standards in training, equipment, and staffing of ICUs, common ethical principles, as along with adherence to international guidelines within the different hospitals, have previously been reported [35]. In addition, we are aware that the SAPS II is not validated for TBI patients, and that several prognostic models exist to predict outcome after TBI (e. g., IMPACT [36] or CRASH [37]). Owing to the design of our database and the information available, we were not able to use these models for this study.

In summary, our results suggest that tracheostomy in patients with moderate or severe isolated TBI might be beneficial, especially if the procedure is performed in the second or third week after admission. The exact reasons for this difference in outcome could be multifactorial and require further investigation.
